# Suicide risk among spouses of patients with dementia: a population-based cohort study

**DOI:** 10.1093/geroni/igaf111

**Published:** 2025-10-13

**Authors:** Hang-Ju Yang, Yu-Han Huang, Wan-Ju Cheng

**Affiliations:** Department of Emergency Medicine, Jen-Ai Hospital Dali Branch, Taichung, Taiwan; Department of Management Office for Health Data, China Medical University Hospital, Taichung, Taiwan; College of Medicine, China Medical University, Taichung, Taiwan; National Center for Geriatrics and Welfare Research, National Health Research Institutes, Miaoli County, Taiwan; Department of Public Health, China Medical University, Taichung, Taiwan

**Keywords:** Care burden, Socioeconomic status, Healthcare service utilization, Major neurocognitive disorder

## Abstract

**Background and Objectives:**

Caregiver burden among spouse caregivers is associated with mental health burdens, including suicide. However, longitudinal studies on suicide risk among spouses of dementia patients are limited. This study aimed to investigate suicide risk among spouses of patients with dementia and to examine how sociodemographic factors and healthcare service utilization influence this risk.

**Research Design and Methods:**

We conducted a population-based cohort study using the 2008–2021 National Health Insurance Research Database (28,696 dementia patient–spouse dyads and matched non-dementia patient–spouse dyads). Dementia and suicide behaviors were identified using diagnostic codes from the national registry. Incident rates of suicide among patients’ spouses were calculated following the diagnosis of dementia. A Cox proportional hazards model assessed suicide risk among spouses of dementia patients relative to spouses of non-dementia patients, stratified by sociodemographic characteristics. We also examined the association between healthcare service utilization by patients with dementia and suicide behavior among their spouses.

**Results:**

Overall suicide risk was similar between spouses of dementia patients and those of non-dementia patients. However, among those in the lowest insurance premium group, spouses of patients with dementia had a 3.2-fold higher (95% confidence interval [CI]: 1.3–8.0) risk of suicide compared to spouses of patients without dementia. The incidence rate ratio of suicide decreased following the diagnosis of dementia but rebounded 10 years after diagnosis. Healthcare utilization was higher among patients with dementia compared to those without dementia, and patient hospitalization was associated with a 2.6-fold increase (95% CI: 1.3–5.3) in suicide risk among their spouses.

**Discussion and Implications:**

The increased suicide risk among spouses of patients with dementia in the later stages of the disease may be related to the financial burden caused by healthcare service utilization. Affordable long-term care services for spouses of patients with dementia should be developed.

Innovation and Translational Significance Spousal caregivers of patients with dementia have been reported to have a high prevalence of suicide, yet it remains unclear whether the risk increases after diagnosis and which social factors contribute. This study found that spouses of patients with dementia with low socioeconomic status had an increased risk of suicide. Additionally, patient hospitalization and late-stage dementia elevated the suicide risk among spouses of patients with dementia. The results underscore the importance of affordable and accessible long-term care services to ease the financial strain on spousal caregivers, especially in the late stages of dementia.

## Background and objectives

As populations age, the caregiving burden has increased substantially. Conditions that demand intensive care—such as severe dementia, multiple comorbidities, or significant functional dependence—are strongly linked to adverse mental health outcomes in caregivers (Huo et al., 2025; Kong & Park, 2022). Among these, dementia care warrants particular public attention because of the growing number of patients diagnosed in developed countries (GBD Dementia Forecasting Collaborators, 2022) and the frequent coexistence of multiple comorbidities. Caregiving for individuals with dementia can be either formal or informal, with the majority of care provided by unpaid family members (i.e., informal) (Kayaalp et al., 2021). It has been estimated that unpaid dementia caregiving in the United States was valued at $346.6 billion in 2023, based on the hours of care provided (Alzheimer’s Association, 2024). Among family members, spouses of individuals with dementia are typically the primary caregivers, especially female spouses. Spouse caregivers have significantly higher levels of caregiving burden than adult children (Liu, 2021), are more likely to live with them, spend more time providing care, and grieve the loss of their partner relationship and intimacy (Ott, 1977; Sanders & Corley, 2003), which further contributes to adverse mental health. Evidence regarding spouse caregivers and their health has been inconsistent in different countries. Spouse caregivers reported poorer personal health and lower social support compared to adult children caregivers in a Korean study (Hong & Kim, 2008), but not in the United States and Spain (Conde-Sala et al., 2010; Rigby et al., 2019).

Caregiving burden has been observed to be associated with mental health problems among informal caregivers, including depression, social isolation, anxiety, and suicide (Amar et al., 2022; Schulz & Sherwood, 2008; Torres et al., 2024). In a meta-analysis involving 1,209 informal caregivers of individuals with dementia (mean age: 63.9 years; 74% women), the prevalence of suicidal ideation was estimated at 32%, whereas suicide attempts ranged from 6% to 16%, although heterogeneity across studies was high (Solimando et al., 2022). In contrast, cross-national estimates in the general population show a lifetime prevalence of suicidal ideation and attempts at 9.2% and 2.7%, respectively (Nock et al., 2008), whereas the 12-month prevalence rates are 2.3% and 0.5% (Cabello et al., 2020). Among adults aged 65 years and older, the 12-month prevalence of suicidal ideation and attempts is 3.7% and 0.6%, respectively (Cabello et al., 2020). Suicidal ideation was more prevalent among female caregivers, those who were unemployed, and individuals with mood disorders (Huo et al., 2025). Beyond individual characteristics of caregivers, social determinants of health among dementia caregivers—such as economic stability, access to healthcare, and community support—have been proposed as important influences (Gaugler et al., 2023).

Because dementia is a chronic, debilitating condition, patients and their families suffer up to 15 years of a disease course of functional decline and increased health service utilization before the patient’s death (Wolters et al., 2019). The long disease course brings a heavy financial burden related to healthcare and long-term care services in addition to the psychological burden. In the United States, families shoulder 64% of the total expenditures for community residents with dementia (Kelley et al., 2020). In Taiwan, informal care costs accounted for the greatest share (42%) of the total cost of care for patients with dementia, and the cost increased with disease severity (Ku et al., 2016). Additional costs include copayments for formal long-term care services, as well as for outpatient visits, emergency room visits, and hospitalizations covered under the Taiwanese National Health Insurance program, which are higher than those incurred by caregivers of people living without dementia (Chan et al., 2022). The caregiving burden may be greater in rural areas, given that long-term care services are more frequently accessed by individuals residing in urbanized regions (Yu et al., 2020). Studies have shown that financial dissatisfaction has been linked to greater levels of depression (Savela et al., 2022), which could trigger stress among caregivers and increase their risk of suicide.

Although the current literature has shown a high prevalence of suicidal ideation or attempts by family caregivers of individuals with dementia, whether dementia poses additional suicide risk to patients’ spouses compared to other diseases has remained unexplored. Furthermore, as long-term care policies for dementia continue to be developed and modified, evidence is needed to examine whether socioeconomic status and health service utilization—both of which contribute to a high financial burden—are associated with an increased suicide risk among spouses of individuals with dementia. This study utilized population-wide healthcare registration data and aimed to examine (1) the risk of suicide behaviors of spouses of patients with dementia compared to spouses of patients without dementia across the disease course, and (2) the association of healthcare service utilization and socioeconomic status with suicide behaviors among spouses of patients with dementia.

## Research design and methods

### Study design

We used Taiwan’s National Health Insurance Research Database (NHIRD) for 2008–2021. The National Health Insurance program has been implemented in Taiwan since March 1995; currently, more than 99% of the Taiwanese population is enrolled in it (Lin et al., 2018). This study was exempt from informed consent because the analysis was of secondary data, and the study was approved by the Institutional Review Board of the Research Ethics Committee of the National Health Research Institutes (EC1130409-E).

### Participants

**Figure 1.**
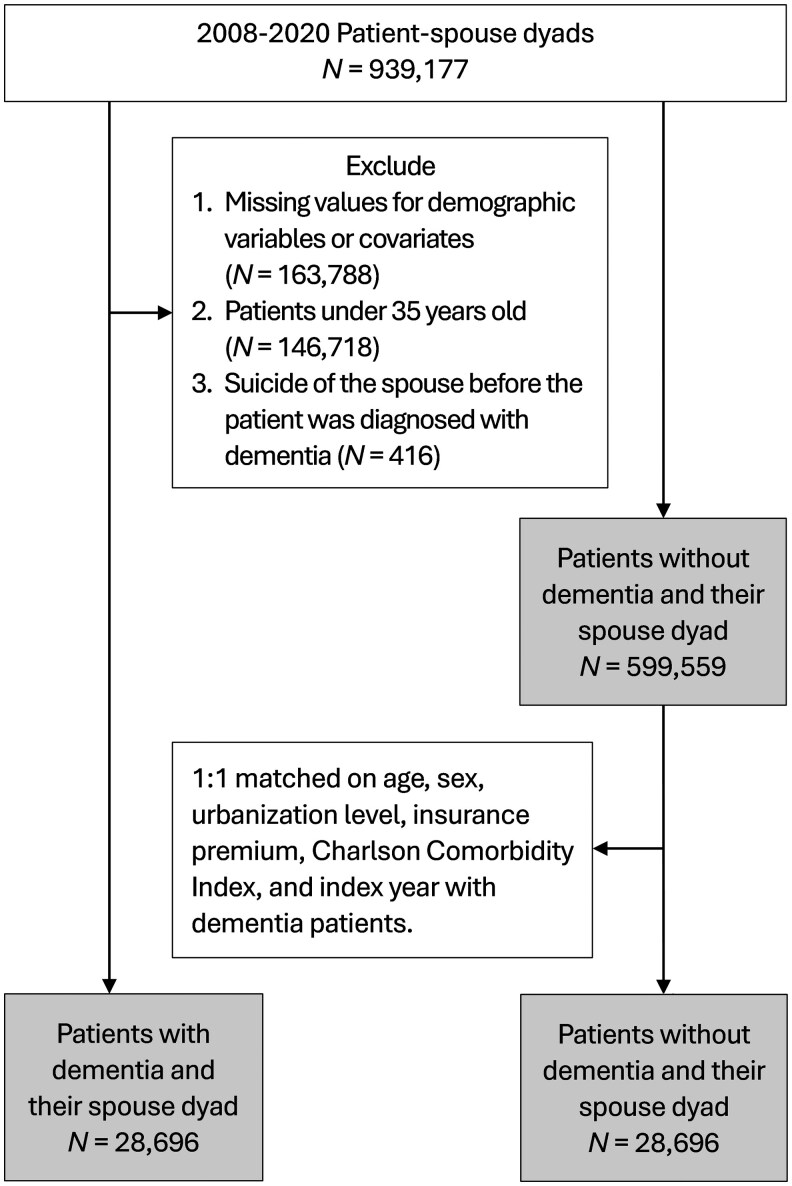
Sample selection flow chart.

In order to identify spouses of patients, we first identified patient–spouse dyads according to insurance premium identity. In Taiwan, the insurance premium is calculated based on the occupation of the index person. Family members of the index person, including spouse, underage children, and unemployed parents, were linked to the index person in the insurance system. Insurance premium was calculated based on income; therefore, in the patient–spouse dyads, one of them must have had income from work at the time of recruitment to be included in this study. Patient–spouse dyads were excluded if both individuals were diagnosed with dementia. We identified 939,177 patient–spouse dyads from the NHIRD from 2008 to 2020 (Figure 1). Among them, we further excluded dyads with missing values for demographic variables and covariates (*N* = 163,788), those in which either individual was under 35 years old (*N* = 146,718), and cases where the spouse’s suicide behaviors occurred before the dementia diagnosis (*N* = 416). After these exclusions, a total of 628,555 eligible patient–spouse dyads remained. Among them, 28,696 patients with dementia and their spouses were identified as the case group. We then randomly selected patients without a diagnosis of dementia from the remaining 599,559 patient–spouse dyads, matching them to the dementia cases on age, sex, urbanization level of the city of residence, insurance premium, Charlson Comorbidity Index (CCI), and index year (i.e., the year of a medical record other than dementia). An additional 28,696 dyads were thus selected as the non-dementia group. The distribution of chronic illnesses (as defined by the CCI) among patients with dementia and patients without dementia is presented in Supplementary Table 1 (see online supplementary material).

### Outcome variables

Suicide behaviors of spouses were identified according to diagnostic codes by physicians in the NHIRD. The International Classification of Diseases, Ninth Revision (ICD-9-CM) codes E950–958 designate “suicide and self-inflicted injuries.” The diagnostic codes did not distinguish between attempted and completed suicide; therefore, both were included as outcomes. The follow-up ended if the spouse emigrated or by December 2021, whichever came first. To account for the competing risk of death, follow-up was further censored at the time of the spouses’ death, identified in the NHIRD, if death occurred before December 2021.

### Socioeconomic status

Insurance premiums and the urbanization level of residence at baseline were selected as proxy measures of socioeconomic position. The monthly insurance premium for the National Health Insurance program was calculated based on the insured income reported by employers, with the premium shared among the government, employers, and employees at varying rates depending on occupational category (Huang et al., 2007). In this study, the monthly insurance premium in New Taiwan Dollars (NTD) was categorized into three groups: $0 to $19,999, $20,000 to $34,999, and more than $34,999. The urbanization index was categorized into three groups: high (metropolitan cities), medium (small cities and suburban areas), and low (rural areas) (Yang et al., 2023).

### Healthcare service utilization

Healthcare utilization among patients with dementia during the follow-up period included hospitalization, emergency room visits, outpatient department visits, and psychiatric department visits (hospitalization or outpatient department visits). The variables were dichotomized as “yes” if any visit occurred during the follow-up period and “no” if no such visit was recorded.

### Covariates

To consider the impact of physical health status on suicide, we calculated the CCI, which is the sum of the weighted scores of 18 comorbid conditions: myocardial infarction, heart failure, peripheral vascular disease, cerebrovascular disease, chronic pulmonary disease, rheumatologic disease, peptic ulcer disease, mild liver disease, hemiplegia or paraplegia, renal disease, malignancy, moderate or severe liver disease, metastatic solid tumor, acquired immune deficiency syndrome, leukemia, lymphoma, and diabetes with and without chronic complication (Charlson et al., 1987). Furthermore, we identified depression (ICD-9: 296.2, 296.3, 296.82, 300.4) and mental disorders other than depression (ICD-9: 291–298, 300–316, excluding 296.2, 296.3, 296.82, and 300.4) of the spouses. Mental disorders were defined as receiving a primary diagnosis in at least three outpatient visits or one inpatient care record in the NHIRD.

### Statistical analysis

Sociodemographic characteristics of spouses of patients with and without dementia were compared using chi-square tests. Due to the large sample size, we calculated the standardized mean difference (SMD) to show the effect size of the difference. The hazard ratio of dementia for suicide among spouses was estimated using the Cox proportional hazard model, adjusted for age, sex, urbanization level of residence, insurance premium, CCI, and mental comorbidities of the spouse. The analysis was stratified by socioeconomic status, that is, urbanization level of residence and insurance premium. The incidence rate ratios (IRRs) of suicide between spouses of patients with and without dementia every 3 years following diagnosis (or index date for patients without dementia) were examined using Poisson regression models.

We examined the differences in health service utilization between patients with and without dementia. Among patients with dementia, the association between health service utilization of the patient during the follow-up period and suicide of the spouses was examined using logistic regression analysis. Each type of utilization—namely, outpatient visits, hospitalizations, emergency room visits, and psychiatric visits—was examined in a separate model. The model was adjusted for age, sex, urbanization level of residence, insurance premium, CCI, and mental comorbidities of the spouse. Data analyses were completed with SAS version 9.4 (SAS Institute).

## Results

Age, sex, and socioeconomic status of spouses of patients with and without dementia were similar at baseline (all SMD <0.1, Table 1). The incidence rate for suicide behaviors per 1,000 person-years was 0.27 among spouses of patients without dementia and 0.31 among spouses of patients with dementia (Table 2). In Cox regression analysis adjusted for age, sex, socioeconomic status, and comorbidities, spouses of patients with dementia did not have a higher risk of suicide than spouses of patients without dementia. Nevertheless, in the analysis stratified by socioeconomic status, spouses of patients with dementia in the low insurance premium group showed a 3.2-fold increased suicide risk compared with spouses of patients without dementia (hazard ratio [HR] = 3.2, 95% confidence interval [CI] = 1.3, 8.0; *p *= .01). The demographic characteristics of this social group are shown in Supplementary Table 2 (see online supplementary material).

**Table 1.** Characteristics of spouses of patients with dementia and without dementia.

Characteristics	Spouses of patients with dementia (*n *= 28,696)		Spouses of patients without dementia (*n *= 28,696)		*p*	Standardized mean difference
	*n*	(%)	*n*	(%)		
**Age (years)**					.001	
** 35–64**	5,996	(20.89)	6,350	(22.13)		0.03
** 65–74**	10,253	(35.73)	10,189	(35.51)		0.01
** ≥75**	12,447	(43.38)	12,157	(42.36)		0.02
**Sex**					.80	
** Male**	16,390	(57.12)	16,360	(57.01)		0.002
** Female**	12,306	(42.88)	12,336	(42.99)		0.002
**Urbanization level**					.67	
** High**	11,917	(41.53)	12,022	(41.89)		0.001
** Medium**	10,204	(35.56)	10,139	(35.33)		0.01
** Low**	6,575	(22.91)	6,535	(22.77)		0.01
**Insurance premium (New Taiwan Dollars)**						
** 0–19,999**	11,394	(39.71)	11,414	(39.78)		0.01
** 20,000–34,999**	15,836	(55.19)	15,763	(54.93)		0.004
** ≥35,000**	1,466	(5.11)	1,519	(5.29)		0.01
**Charlson Comorbidity Index**					.02	
** 0**	18,218	(63.49)	18,289	(63.73)		0.02
** 1**	4,359	(15.19)	4,133	(14.40)		0.03
** ≥2**	6,119	(21.32)	6,274	(21.86)		0.002
**Mental comorbidities**						
** Depression**	165	(0.57)	118	(0.41)	.005	0.03
** Other than depression**	357	(1.24)	320	(1.12)	.15	0.01

**Table 2.** Stratification analysis for suicide in spouses of patients with and without dementia by sociodemographic characteristics of spouses.

Variable	Spouses of patients without dementia			Spouses of patients with dementia			Cox proportional hazard models					
							Crude model			Adjusted model^a^		
	*N*	PY	IR	*N*	PY	IR	HR	(95% CI)	*p*	HR	(95% CI)	*p*
**All participants**	39	142,767	0.27	40	130,319	0.31	1.20	(0.77, 1.86)	.43	1.18	(0.76, 1.83)	.47
**Urbanization level**												
** High**	12	63,490	0.19	17	55,226	0.31	1.74	(0.83, 3.66)	.14	1.69	(0.81, 3.56)	.16
** Medium**	18	52,103	0.35	15	46,150	0.33	1.05	(0.53, 2.08)	.90	1.05	(0.53, 2.10)	.88
** Low**	9	32,671	0.28	8	28,943	0.28	1.10	(0.42, 2.87)	.84	1.05	(0.40, 2.75)	.92
**Insurance premium (New Taiwan Dollars)**												
** 0–19,999**	7	63,080	0.11	16	52,425	0.31	3.01	(1.23, 7.34)	.02	3.23	(1.31, 7.97)	.01
** 20,000–34,999**	32	77,627	0.41	24	71,098	0.34	0.88	(0.52, 1.50)	.65	0.85	(0.50, 1.44)	.54
** ≥ 35,000**	0	7,558	0.00	0	6,796	0.00	—		—	—		—

*Note.* CI = confidence interval; HR = hazard ratio; IR = incidence rate (per 1,000 person-years); PY = person-year.

a The adjusted Cox proportional hazard models account for sex, age, urbanization level, insurance premiums, and mental and physical comorbidities, where the reference group is spouses of patients without dementia.

**Figure 2.**
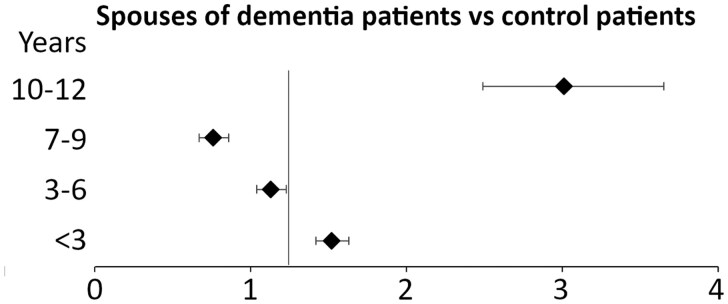
Incidence rate ratios (IRRs) for suicide among spouses of patients with dementia compared to spouses of patients without dementia by 3-year periods following the diagnosis of dementia. ^a^The adjusted Poisson models account for sex, age, urbanization level, insurance premiums, and mental and physical comorbidities.

Across the disease course (Figure 2 and Supplementary Table 3 [see online supplementary material]), the IRR for suicide among spouses of patients with dementia compared to spouses of patients without dementia had a 1.5-fold suicide risk (95% CI = 1.4, 1.6; *p *< .001) within the first 3 years of dementia diagnosis. The IRR decreased between 3 and 9 years following the diagnosis but increased again to 3-fold 10 years following diagnosis (95% CI = 2.5, 3.7; *p *< .001).

Regarding healthcare service utilization (Table 3), a higher proportion of patients with dementia had outpatient visits (99.7% vs 90.6%), hospitalization (50.8% vs 35.6%), ER visits (71.6% vs. 52.8%), and psychiatric visits (41.6% vs 8.9%), compared to patients without dementia. Additionally, patient hospitalization was associated with a 2.6-fold suicide risk among spouses of patients with dementia (odds ratio [OR] = 2.6, 95% CI = 1.3, 5.3). Emergency room visits, psychiatric visits, and outpatient visits were not associated with suicide risks.

**Table 3.** Healthcare utilization among patients with dementia and the suicide risk of their spouses.

Healthcare utilization of patients with dementia	Patients with dementia (*N *= 28,696)		Patients without dementia (*N *= 28,696)		*p*	Standardized mean difference	Logistic regression models predicting suicide behaviors of the spouses					
							Crude model			Adjusted model^a^		
	*N*	(%)	*N*	(%)			OR	(95% CI)	*p*	OR	(95% CI)	*p*
**Outpatient visits**	28,608	(99.69)	25,985	(90.55)	<.001	0.434	1.00	(1.00, 1.00)	0.99	1.00	(1.00, 1.00)	.99
**Hospitalization**	14,569	(50.77)	10,221	(35.62)	<.001	0.310	2.56	(1.28, 5.13)	0.01	2.62	(1.30, 5.27)	.01
**Emergency room visits**	20,538	(71.57)	15,164	(52.84)	<.001	0.394	2.25	(0.95, 5.37)	0.07	2.24	(0.94, 5.36)	.07
**Psychiatric visits**	11,925	(41.56)	2,543	(8.86)	<.001	0.813	0.84	(0.45, 1.60)	0.60	0.86	(0.45, 1.64)	.65

*Note.* CI = confidence interval; OR = odds ratio.

a The logistic regression models included only dementia patient–spouse dyads, with the patients’ healthcare utilization used to predict suicide behaviors in their spouses. Each type of utilization—namely, outpatient visits, hospitalizations, emergency room visits, and psychiatric visits—was examined in a separate model. All adjusted models controlled for sex, age, urbanization level, insurance premium, and mental and physical comorbidities.

## Discussion and implications

In this longitudinal cohort, spouses of patients with dementia with disadvantaged financial status are at increased risk of suicide compared to spouses of patients without dementia. The suicide risk decreased following diagnosis but rebounded 10 years after the diagnosis of dementia. Healthcare service utilization was high among patients with dementia, and patient hospitalization was associated with an increased risk of suicide among their spouses.

In this study, the overall suicide risk observed among both spouses of patients with and without dementia highlights the need for greater attention to the mental health of spousal caregivers. Our novel finding is that the dementia diagnosis of the patient was associated with increased risk of suicide in spouses only in the group with disadvantaged financial status, highlighting the burden brought about by dementia in this social group. This social group was characterized by old age and a high proportion of female spouses with high comorbidity burden (Supplementary Table 2 [see online supplementary material]), underscoring an accumulation of disadvantages. Notably, the 95% confidence interval of the hazard ratio estimated by the Cox proportional hazards model was wide, likely due to the small number of suicide cases in this group. Replication in larger samples or different populations is warranted in future studies. Previous studies have observed an association between low income and caregivers’ depressive symptoms and perceived caregiver burden for patients with dementia (Chiao et al., 2015; Covinsky et al., 2003; Ku et al., 2019), which may increase the risk of suicide. In this study, we observed a higher prevalence of depression among spouses of patients with dementia compared to spouses of patients without dementia. Nevertheless, the increased suicide risk remained after adjustment for depressive disorders, suggesting that in addition to screening and treating depressive disorders of spouses, resource support may also be crucial.

The increased suicide risk among spouses of patients with dementia diminished in the years following diagnosis, likely as a result of psychological adaptation to the bereavement process (de Groot & Kollen, 2013; Zisook & Shuchter, 1985). ­However, we observed that the suicide risk was highest after 10 years of dementia diagnosis, when the patients are in the late stage of the disease and need full-time care (Chin et al., 2024). With the progression of the disease course, the cost of care doubles compared to mild dementia (Ku et al., 2016). Additionally, hospitalization, but not psychiatric or ER visits, is associated with increased risk of suicide in spouses of patients with dementia in this study. Hospitalization may indicate greater disease severity and more comorbidities, leading to higher costs. An earlier study in Taiwan showed that functional decline is associated with care costs for patients with dementia, but neuropsychiatric symptoms are not (Ku et al., 2019). These observations suggest that neuropsychiatric symptoms with available healthcare service did not elevate caregivers’ suicide risk. However, hospitalization is more costly than other healthcare services. Furthermore, long-term care services in Taiwan are under development, but their costs remain higher than outpatient visits, ER visits, and hospitalization services, which are covered by the National Health Insurance program (Chan et al., 2022; Chiu & Shyu, 2001). The elevated risk of suicide observed 10 years after a dementia diagnosis may reflect the chronic stress and prolonged burden experienced by caregivers. A specific form of suicide among caregivers, accompanied by the homicide of the patients, has been identified (Karch & Nunn, 2011) and may be related to inadequacies in the long-term care system. A study in Korea reported cases of companion suicides of caregivers following the homicide of the patient with dementia (Kim, 2014). In Japan, the annual prevalence of homicide of care recipients by caregivers, with violent means, seems to be increasing, reflecting exhaustion of caregivers (He et al., 2009). Narratives of caregivers of patients with dementia showed the idea of death, either that of the patient or the caregiver themselves, as a perceived means of escaping the caregiving burden, especially after many years of care (Anderson et al., 2019; Zhang et al., 2023). It is therefore important to screen the mental health of spouses of patients with dementia and to provide integrated resource support, particularly for those with disadvantaged socioeconomic status, during healthcare and long-term care encounters in order to help prevent such tragedies.

To our knowledge, this is the first study to utilize nationwide registry data to examine suicide among spouses of individuals with dementia. Our findings are strengthened by the longitudinal design, physician-diagnosed dementia and suicide, and registered healthcare utilization data. Nevertheless, this study has several limitations. First, we were unable to determine whether the spouse of a patient with dementia was the primary caregiver or bore the financial burden. Otherwise, spouses of patients may suffer the loss of an intimate relationship or loss of their social activities, which may contribute to suicide risk. Second, our analysis, based on medical claim data, did not consider several factors that may confound the association, including the clinical severity of dementia, its impact on quality of life, and spouses’ personal psychological characteristics. Additionally, education access, economic stability, healthcare quality, and the availability of family and community support may differ between spouses of patients with dementia and those of patients with other chronic illnesses, potentially confounding the associations observed in this study. Future research is warranted to further explore the social determinants of caregivers’ health in order to inform policies and public health actions aimed at supporting dementia caregivers (Gaugler et al., 2023). Third, the study participants were limited to married patient–spouse dyads identified through the insurance premium registry. Therefore, the results are not generalizable to couples not identified by this method, such as those who cohabit without marriage registration or those without a recorded work history to generate insurance premiums.

In conclusion, this study found an increased suicide risk only among spouses of patients with dementia who were in disadvantaged financial circumstances, with the risk peaking when the patient reached the late stage of the disease. This heightened suicide risk may be related to increased disease severity and caregiving stress and the financial burden associated with greater healthcare demands during this stage, while the spouses’ own aging-related physical and functional health problems may further contribute. Therefore, in addition to psychoeducational interventions for caregivers in reducing caregiver distress (Adelman et al., 2014), resource allocation and affordable long-term care services for spouses of patients with dementia are crucial.

## Supplementary Material

igaf111_Supplementary_Data

## Data Availability

Due to legal restrictions, data cannot be made publicly available. Requests for data can be sent as a formal proposal to Taiwan’s Health and Welfare Data Science Centre (https://dep.mohw.gov.tw/dos/mp-113.html). This study has not been preregistered.
