# Supplementary material for: Suicide risk among spouses of patients with dementia: a population-based cohort study
Source: Innov Aging. 2025 Oct 13;9(11):igaf111. doi: 10.1093/geroni/igaf111 (PMC12640235; doi:10.1093/geroni/igaf111)
Supplement: igaf111_Supplementary_Data [file igaf111_supplementary_data.zip › innage suppl Yang, Huang & Cheng.docx]

***Innovation in Aging* Supplementary Material: Yang, Huang & Cheng.** **Suicide Risk Among Spouses of Patients with Dementia: A Population-Based Cohort Study.**

**Supplementary Table 1.** Distribution of chronic illnesses in patients with dementia and without dementia.

| **Chronic illnesses** | **Patients with dementia (*n*=28,696)** | | **Patients without dementia  (*n*=28,696)** | | ***p*** | **Standardized mean difference** |
| --- | --- | --- | --- | --- | --- | --- |
|  | ***n*** | **(%)** | ***n*** | **(%)** |  |  |
| Myocardial infarction | 881 | (3.07) | 919 | (3.20) | 0.36 | 0.008 |
| Congestive heart failure | 2,136 | (7.44) | 1,764 | (6.15) | <0.001 | 0.052 |
| Peripheral vascular disease | 488 | (1.70) | 435 | (1.52) | 0.08 | 0.015 |
| Cerebrovascular disease | 6,929 | (24.15) | 4,360 | (15.19) | <0.001 | 0.227 |
| Chronic pulmonary disease | 2,813 | (9.80) | 2,562 | (8.93) | <0.001 | 0.030 |
| Rheumatologic disease | 354 | (1.23) | 360 | (1.25) | 0.82 | 0.002 |
| Peptic ulcer disease | 4,049 | (14.11) | 3,692 | (12.87) | <0.001 | 0.036 |
| Mild liver disease | 2,876 | (10.02) | 2,855 | (9.95) | 0.77 | 0.002 |
| Diabetes without chronic complication | 1,939 | (6.76) | 1,334 | (4.65) | <0.001 | 0.091 |
| Diabetes with chronic complication | 7,416 | (25.84) | 6,351 | (22.13) | <0.001 | 0.087 |
| Hemiplegia or paraplegia | 1,771 | (6.17) | 1,128 | (3.93) | <0.001 | 0.102 |
| Renal disease | 1,865 | (6.50) | 1,402 | (4.89) | <0.001 | 0.070 |
| Malignancy other than leukemia and lymphoma | 2,829 | (9.86) | 4,810 | (16.76) | <0.001 | 0.204 |
| Leukemia | 34 | (0.12) | 88 | (0.31) | <0.001 | 0.041 |
| Lymphoma | 70 | (0.24) | 89 | (0.31) | 0.13 | 0.013 |
| Moderate or severe liver disease | 246 | (0.86) | 270 | (0.94) | 0.29 | 0.009 |
| Metastatic solid tumor | 22 | (0.08) | 74 | (0.26) | <0.001 | 0.044 |
| AIDS/HIV | 4 | (0.01) | 4 | (0.01) | >0.99 | <0.001 |

**Supplementary Table 2.** Characteristics of spouses of patients with and without dementia in the lowest insurance premium group.

| **Characteristics** | **Spouses of patients with dementia (*n*=11,394)** | | **Spouses of patients without dementia  (*n*=11,414)** | | ***p*** | **Standardized mean difference** |
| --- | --- | --- | --- | --- | --- | --- |
|  | ***n*** | **(%)** | ***n*** | **(%)** |  |  |
| Age (years) |  |  |  |  | 0.46 |  |
| 35–64 | 1,432 | (12.57) | 1,493 | (13.08) |  | 0.015 |
| 65–74 | 3,456 | (30.33) | 3,472 | (30.42) |  | 0.002 |
| ≥ 75 | 6,506 | (57.10) | 6,449 | (56.50) |  | 0.012 |
| Sex |  |  |  |  | 0.60 |  |
| Male | 2,870 | (25.19) | 2,841 | (24.89) |  | 0.007 |
| Female | 8,524 | (74.81) | 8,573 | (75.11) |  | 0.007 |
| Urbanization level |  |  |  |  | 0.94 |  |
| High | 6,055 | (53.14) | 6,065 | (53.14) |  | <0.001 |
| Medium | 3,755 | (32.96) | 3,745 | (32.81) |  | 0.003 |
| Low | 1,584 | (13.90) | 1,604 | (14.05) |  | 0.004 |
| Charlson Comorbidity Index |  |  |  |  | <0.001 |  |
| 0 | 6,897 | (60.53) | 6,786 | (59.45) |  | 0.022 |
| 1 | 1,787 | (15.68) | 1,631 | (14.29) |  | 0.039 |
| ≥ 2 | 2,710 | (23.78) | 2,997 | (26.26) |  | 0.057 |
| Mental comorbidities |  |  |  |  |  |  |
| Depression | 77 | (0.68) | 41 | (0.36) | 0.001 | 0.04 |
| Other than depression | 144 | (1.26) | 135 | (1.18) | 0.58 | 0.007 |

**Supplementary Table 3.** Incidence rate ratios (IRR) for suicide among spouses of patients with dementia compared to spouses of patients without dementia by 3-year periods following the diagnosis of dementia.

| **Year from the index year** | **Crude IRR (95%CI)** | ***p*** | **Adjusted IRR (95%CI) ^a^** | ***p*** |
| --- | --- | --- | --- | --- |
| <3 | 1.55 (1.45, 1.66) | <0.001 | 1.52 (1.42, 1.63) | <0.001 |
| 3–6 | 1.13 (1.03, 1.23) | 0.01 | 1.13 (1.04, 1.23) | 0.01 |
| 7–9 | 0.73 (0.65, 0.83) | <0.001 | 0.76 (0.67, 0.86) | <0.001 |
| 10–12 | 2.62 (2.16, 3.18) | <0.001 | 3.01 (2.49, 3.65) | <0.001 |

Note. 95% CI = 95% confidence interval.

^a^ Models were adjusted for age, sex, socioeconomic status, and comorbidities .
